# Untargeted Metabolic Profiling of 4-Fluoro-Furanylfentanyl and Isobutyrylfentanyl in Mouse Hepatocytes and Urine by Means of LC-HRMS

**DOI:** 10.3390/metabo11020097

**Published:** 2021-02-10

**Authors:** Camilla Montesano, Flaminia Vincenti, Federico Fanti, Matteo Marti, Sabrine Bilel, Anna Rita Togna, Adolfo Gregori, Fabiana Di Rosa, Manuel Sergi

**Affiliations:** 1 Department of Chemistry, Sapienza University of Rome, 00185 Rome, Italy; flaminia.vincenti@uniroma1.it; 2 Department of Public Health and Infectious Disease, Sapienza University of Rome, 00185 Rome, Italy; 3 Faculty of Bioscience and Technology for Food, Agriculture and Environment, University of Teramo, 64100 Teramo, Italy; ffanti@unite.it (F.F.); msergi@unite.it (M.S.); 4 Department of Translational Medicine, Section of Legal Medicine and LTTA Centre, University of Ferrara, 44121 Ferrara, Italy; sabrine.bilel@unife.it; 5 Department of Anti-Drug Policies, Collaborative Center for the Italian National Early Warning System, Presidency of the Council of Ministers, University of Ferrara, 44121 Ferrara, Italy; 6 Department of Physiology and Pharmacology Vittorio Erspamer, Sapienza University of Rome, 00185 Rome, Italy; annarita.togna@uniroma1.it; 7 Carabinieri, Department of Scientific Investigation (RIS), 00191 Rome, Italy; adolfo.gregori@carabinieri.it (A.G.); Fabiana.dirosa@carabinieri.it (F.D.R.)

**Keywords:** fentanyl analogs, metabolic profile, liquid chromatography-high resolution mass spectrometry, in vitro and in vivo metabolism, new psychoactive substances

## Abstract

The diffusion of new psychoactive substances (NPS) is highly dynamic and the available substances change over time, resulting in forensic laboratories becoming highly engaged in NPS control. In order to manage NPS diffusion, efficient and innovative legal responses have been provided by several nations. Metabolic profiling is also part of the analytical fight against NPS, since it allows to identify the biomarkers of drug intake which are needed for the development of suitable analytical methods in biological samples. We have recently reported the characterization of two new analogs of fentanyl, i.e., 4-fluoro-furanylfentanyl (4F-FUF) and isobutyrylfentanyl (iBF), which were found for the first time in Italy in 2019; 4F-FUF was identified for the first time in Europe and was notified to the European Early Warning System. The goal of this study was the characterization of the main metabolites of both drugs by in vitro and in vivo experiments. To this end, incubation with mouse hepatocytes and intraperitoneal administration to mice were carried out. Samples were analyzed by means of liquid chromatography-high resolution mass spectrometry (LC–HRMS), followed by untargeted data evaluation using Compound Discoverer software with a specific workflow, designed for the identification of the whole metabolic pattern, including unexpected metabolites. Twenty metabolites were putatively annotated for 4F-FUF, with the dihydrodiol derivative appearing as the most abundant, whereas 22 metabolites were found for iBF, which was mainly excreted as nor-isobutyrylfentanyl. *N*-dealkylation of 4F-FUF dihydrodiol and oxidation to carbonyl metabolites for iBF were also major biotransformations. Despite some differences, in general there was a good agreement between in vitro and in vivo samples.

## 1. Introduction

The use of new psychoactive substances (NPSs), introduced as legal alternatives for controlled drugs [1], has become a worldwide phenomenon since the early 90s and it has been exacerbated by the increase in online selling points and sales [2]. To manage NPS issues, efficient and innovative legal responses against the diffusion of new drugs have been provided by several nations. However, the NPS market is highly dynamic and the available substances change over time, resulting in forensic laboratories becoming highly engaged in the fight against NPSs. From an analytical point of view, NPS detection in both seizures and biological samples is a challenge due to the lack of analytical standards, library spectra and pharmacokinetic information. High resolution mass spectrometry (HRMS) is becoming the technique of choice to deal with NPSs and to overcome the limitations of low resolution MS coupled with liquid or gas chromatography (LC or GC), which are usually used in targeted acquisition modes [3,4]. In fact, accurate mass, contrary to nominal masses, may be used to ascertain the molecular formula and putatively annotate a new molecule, when fragmentation spectra are available [5]. Metabolic profiling is also part of the analytical fight against NPSs, since it allows to identify the biomarkers of drug intake, which are needed for the development of suitable analytical methods in biological samples [6]. Characterization of drug metabolites is usually performed using in vitro and/or in vivo studies, which can be assisted by in-silico prediction tools to make LC-HRMS data analysis easier [7]. In vitro studies involve incubation of the drugs with human or animal hepatocyte cultures [8,9], human liver preparations [10] or the fungus *Caenorhabditis elegans* [11], whereas biological samples collected in authentic cases or samples obtained by controlled drug administration to rats, mice and other rodents may be used for in vivo studies. Novel in vivo models such as zebrafish (*Danio rerio*) larvae have been recently reported as a good alternative for NPS metabolism studies [12]. Conversely, controlled human studies would be best suited but are not practicable for ethical reasons and the lack of preclinical safety data.

Metabolic studies have been carried out for a wide range of NPSs from different classes, including synthetic opioids [9,13]. Among this group, fentanyl, also called “synthetic heroin” [14], and its analogues deserve special attention. In the last years these drugs, originally introduced to treat severe pain, and later produced in illegal laboratories, started to appear in the illicit market to replace scheduled related compounds [15,16,17]. In 2017, about 1300 seizures of new opioids were reported to the EU Early Warning System (EWS) by national law enforcement agencies; 70% of these included fentanyl derivatives, which were often sold as or mixed with heroin [18]. Given the danger of these compounds, a broader knowledge of the metabolic behavior of fentanyl derivatives is mandatory. In recent years, the metabolisms of many of these, including furanylfentanyl [19,20,21]; butyrylfentanyl [22,23]; 4-fluoro-isobutyrylfentanyl [19] and ortho-, meta- and para-fluorofentanyl [24], have been studied. Similarly, to fentanyl, for most of these drugs the *N*-dealkylated metabolites were shown to be the primary biomarkers; however, it was reported that other biotransformations, including phase I mono- and di-hydroxylation, oxidation to carboxylic acid and phase II glucuronidation and sulfation, dominated metabolite formation [25]. Furanyl fentanyl exhibited a rather different behavior, arising from the heterocyclic furane moiety, with amide hydrolysis and dihydrodiol formation being the principal biotransformations.

We have recently reported the characterization of two new analogs of fentanyl, i.e., 4-fluoro-furanylfentanyl (4F-FUF) and isobutyrylfentanyl (iBF), which were reported for the first time in Italy in 2019 [26]; 4F-FUF was identified for the first time in Europe and a notification to the European Early Warning System (EWS) resulted from the previously cited study. iBF is closely related to fentanyl, with a methyl group linked to the α-carbon in the propionyl group, whereas 4F-FUF differs from fentanyl by a furan-2-carboxamide instead of the propionamide group and a fluorine atom in the para position on the aromatic group. Being two new derivatives, to the best of our knowledge their metabolic profile has not been characterized to date, and the pharmacological effects are also unknown; however, dose-dependent increases in locomotion and antinociception were reported for iBF [27]. For iBF, different isomers of the hydroxylated metabolites were characterized in hepatocyte samples; however, the complete metabolic profile was not investigated [28].

The goal of the present study was the characterization of the main metabolites of both drugs by both in vitro and in vivo experiments. To this end, incubation with mouse hepatocytes and intraperitoneal administration in mice were carried out. In vivo experiments were carried out to confirm the in vitro results, as well as to study pharmacotoxicological effects, which will be presented in a future study. Untargeted analysis of the obtained samples was performed using LC-HRMS, whereas Compound Discoverer software was used for data analysis with an untargeted workflow to putatively identify even unexpected metabolites.

## 2. Results and Discussion

In this study the main metabolites of iBF and 4F-FUF were studied through in vitro and in vivo studies. All the samples were analyzed using LC-HRMS in data-dependent acquisition mode, which led to the triggering of MS^2^ events for the most intense precursor ions. With this setting, semi-quantitative information may be obtained from the MS full scan, while the analysis of the MS^2^ spectra may allow one to putatively annotate the detected metabolites. The software Compound Discoverer was selected to automatically extract the metabolic features, using an untargeted approach in order to detect the expected metabolites on the basis of the biotransformations that may occur, as well as the unexpected ones.

Concerning the in vitro study, positive and negative controls served to monitor the incubation. As reported in Section 3. Materials and Methods, diclofenac and testosterone were used as positive controls respectively for phase I and phase II metabolism to ensure that proper incubation conditions were maintained: hydroxydiclofenac was observed in the diclofenac positive control, confirming phase I hepatocyte metabolic activity, whereas testosterone glucuronide and sulphate were detected in the testosterone control, showing that phase II metabolic activity also occurred. On the other hand, all the peaks corresponding to metabolites of iBF and 4F-FUF discussed in the next paragraphs were not detected in the negative controls.

### 2.1. 4F-FUF Metabolic Profile In Vitro and In Vivo

Overall, 20 metabolites were putatively identified for 4F-FUF, 16 were found in both urine and hepatocytes samples, and four of them were only found in urine. In order to highlight the similarities and differences obtained in vitro and in vivo, the results of the two studies will be presented together. A list of all the metabolites with the proposed metabolic transformation and elemental composition, as well as the retention time, accurate mass of the protonated molecule and mass error, are provided in Table 1. The quantitative results of the in vitro and in vivo studies in terms of peak areas in hepatocyte and urine samples (average) are shown in Table 2. The extracted ion currents of the 20 metabolites are shown in Figure S1.

It is worth noting that 4F-FUF showed a pronounced antidiuretic effect in vivo and no samples could be obtained in the first six hours after administration. Syndrome of inappropriate antidiuretic hormone secretion was previously associated with fentanyl consumption [29]; however, the reduction in urine production was not significant following iBF administration, indicating that the distinct chemical structure of 4F-FUF is responsible for this effect.

The parent drug was found in all the samples; after two hours of drug incubation with hepatocytes, the peak area was reduced by nearly 90%, showing an intense metabolic activity. P_FFUF was also found in urine. In the first samples obtained (6–12 h) the peak area represented ≈7% of the sum of the area of all the metabolites found; this relative amount was reduced to ≈2% at the last time point (24–31 h). Relative quantification is limited by probable differences in ionization efficiency for the different metabolites and the parent compound. Another issue is the matrix effect, which can negatively or, less commonly, positively affect the absolute peak areas, but with the matrices being relatively simple, we did not expect an excessive enhancement or suppression of the signals [30].

**Figure 1:**
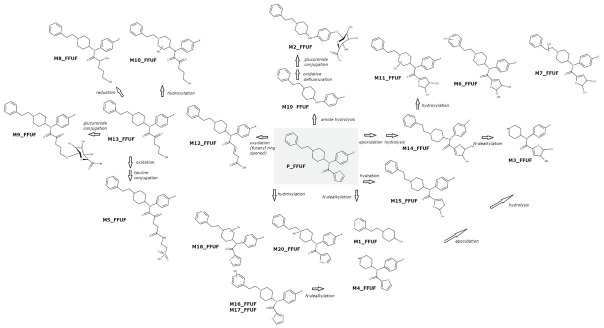
Proposed metabolic pathway of 4-fluoro-furanylfentanyl combining both the in vitro and in vivo studies.

A scheme of the metabolic profile of 4F-FUF is shown in Figure 1. Both in vitro and in vivo, the most intense metabolite, relatively, was M14_FFUF, which corresponded to the dihydrodiol metabolite, resulting from epoxidation of furan, followed by hydration [19]. *N*-dealkylation of M14_FFUF was shown to be another major biotransformation, producing M3_FFUF, which was the second most relatively intense metabolite in vivo; on the contrary, it was only a minor bio-product in the hepatocyte samples. In vitro, *N*-dealkylation of the parent compound was predominant, leading to the nor-4F-FUF metabolite (M4_FFUF), which instead showed a low intensity in urine samples. The dihydrodiol metabolite was also further hydroxylated at the piperidine ring to form M11_FFUF and at the phenylethyl moiety to form M6_FFUF and M7_FFUF, which were quite intense in urine but were not detected in hepatocytes.

Amide hydrolysis leading to despropionyl fentanyl, previously identified as the major metabolite of furanyl fentanyl [19,20,21] which only differs from 4F-FUF for the fluorine atom, appeared to be a minor biotransformation both in vitro and in vivo. This route led to M19_FFUF which was mainly detected in hepatocytes and M2_FFUF which resulted from oxidative defluoruration and glucuronidation of M19_FFUF. The lower prevalence of metabolites deriving from amide hydrolysis in the metabolic profile of 4F-FUF when compared to its defluorinated analogue is not surprising since fluorine substitution can have complex effects on drug metabolism, in terms of route(s) and ex tent; in fact, fluorine substitution even at sites distal to the site of metabolic attack can affect metabolism by either inductive/resonance effects or conformational and electrostatic effects [31]. Hydration of 4F-FUF at the furanyl ring, leading to M15_FFUF, and furanyl ring opening were also observed. Different low-intensity metabolites were formed through this reaction, including M8_FFUF and M13_FFUF, whereas a further hydroxylation or carbonylation led to M10_FFUF and M12_FFUF, respectively. These reactions are typical of furane-containing molecules [32]. Phase II biotransformations were observed for these metabolites: glucuronidation of M13 produced M9_FFUF, whereas an unexpected taurine conjugate of M12_FFUF (M5_FFUF) was found, in a higher amount in urine samples. Hydroxylation at the phenylethyl moiety was a further minor biotransformation. Different isomers (M16_FFUF, M17_FFUF, M18_FFUF), including the *N*-oxide (M20_FFUF), were observed. These metabolites had a relatively low intensity in both hepatocytes and urine. Finally, oxidative *N*-dealkylation led to M1_FFUF, which was a minor metabolite.

### 2.2. Isobutyrylfentanyl Metabolic Profile In Vitro and In Vivo

In total, 22 metabolites of iBF were putatively identified in this study; eight of them were only detected in vivo. iBF exhibited a lower antidiuretic effect compared to 4F-FUF; however, only one sample could be collected one hour after drug administration and two samples were available after two hours. No sample was available after four hours.

**Figure 2:**
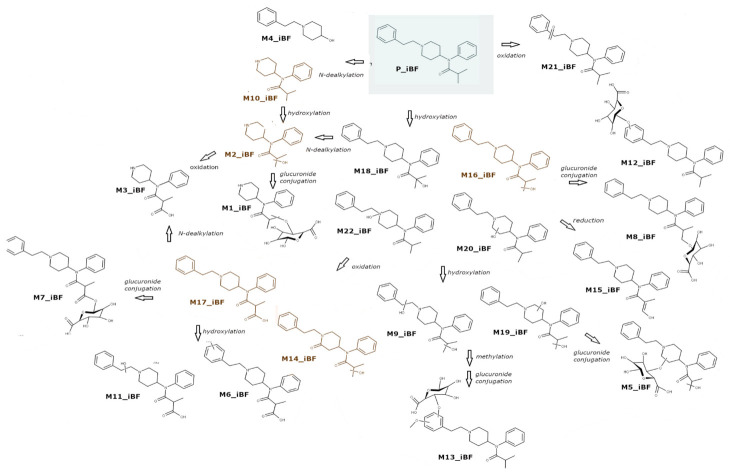
Proposed metabolic pathway of isobutyrylfentanyl combining both the in vitro and in vivo studies.

All the putatively annotated metabolites are listed in Table 3, which also reports the proposed metabolic transformation, the retention time, the accurate mass and elemental composition of the protonated molecule and the mass error. The peak areas in hepatocyte and urine samples (average) are shown in Table 4. Extracted ion currents of the 22 metabolites are shown in Figure S2, whereas a scheme of the metabolic profile of iBF is shown in Figure 2. Similarly, to 4F-FUF, P_iBF was found in all the samples but a pronounced metabolic activity was observed with only 5% of the initial amount found in hepatocytes after 3 h. Nor-iBF (M10_iBF), which was formed by *N*-dealkylation of iBF, was the most intense metabolite both in hepatocytes and in urine. Another major biotransformation was hydroxylation, which led to four isomers, with the -OH group placed at the isobutyryl moiety (M15_iBF, M16_iBF and M18_iBF), or on the phenylethyl aromatic ring (M20_iBF). The *N*-oxide was also detected (M22_iBF); this metabolite is characterized by a higher retention time (Rt) than the parent drug, and these data were in accordance with the literature [19,23]. Hydroxylated metabolites, and especially ω-hydroxy-iBF (M16_iBF), were quite intense in hepatocytes, whereas in urine this was an intermediate metabolite which underwent subsequent biotransformations, producing major metabolites. Oxidation of M16_iBF prevailed in vivo and gave rise to the second and third main metabolites, M17_iBF and M14_iBF respectively. In vitro these metabolites were found in a very low amount, in accordance with the results of Kanamori et al. for butyrylfentanyl [22]; a likely explanation was a low activity of alcohol and aldehyde dehydrogenase in hepatocytes. M17_iBF was ω-carboxy-iBF; further metabolic steps were observed for this metabolite. Hydroxylation produced M6_iBF and M11_iBF, *N*-dealkylation gave rise to M3_iBF, and M7_iBF and M1_iBF were obtained by glucuronide conjugation of M17_iBF and M2_iBF, respectively. *N*-dealkylation of M16_iBF was also observed, leading to M2_iBF, which was intense in urine. Finally, phase II glucuronide conjugation was also possible, with the formation of M8_iBF and M12_iBF.

Other minor metabolites were formed by carbonylation (M21_iBF), oxidative *N*-dealkylation (M4_iBF), dihydroxylation of iBF (M9_iBF and M19_iBF) and subsequent methylation; phase II glucuronidation was observed for these metabolites, producing M5_iBF and M13_iBF respectively.

This is the first report of iBF’s complete metabolic profile; however, it should be pointed out that in a study conducted by Wallgren et al. [28] iBF was included among the investigated drugs, and some hydroxylated metabolites isomers were characterized and quantified in hepatocyte samples. In addition, iBF-related fentanyl analogues, i.e., butyrylfentanyl [17,22,23] and 4F-iBF [19], were previously studied by other authors, both in vitro and in vivo. The detected metabolites were similar; in the cited studies for butyrylfentanyl, it was observed that nor-butyrylfentanyl was only intense in vitro, whereas it was a minor metabolite in vivo, with ω-OH-butyrylfentanyl and ω-carboxy-butyrylfentanyl being the relatively most intense. In these studies the available samples were obtained post-mortem and redistribution was deemed responsible for the reduction of the nor-metabolite. For 4F-iBF, the nor-metabolite was the main metabolite both in vivo and in vitro, similarly to what we found; on the other hand, aliphatic hydroxylation was a minor pathway, whereas the piperidine ring and the phenylethyl moiety were the main biotransformation sites. These divergences show that the fluorine atom may have a significant impact on the biotransformation routes, similarly to what was observed for 4F-FUF and FUF.

### 2.3. Elucidation of Metabolites Structure

**Figure 3:**
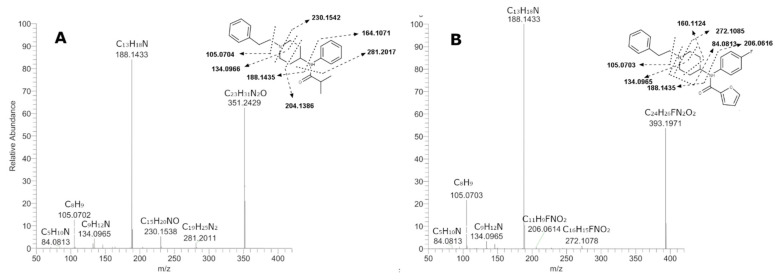
MS/MS fragmentation spectra of isobutyrylfentanyl (**A**) 4-fluoro-furanylfentanyl (**B**) and the postulated fragmentation pattern.

For the elucidation of metabolite structures, an in-depth analysis of the corresponding MS/MS spectra was necessary. Initially, characteristic fragments of the parent drugs were taken into account to determine the biotransformation sites. The spectra and the postulated fragmentation patterns of both 4F-FUF and iBF MS/MS core structures are depicted in Figure 3, which shows for both compounds two main fragments: one at mass-to-charge ratio (*m*/*z*) 105.0703, corresponding to the phenethyl moiety, and one at *m*/*z* 188.1435, resulting from the cleavage between the piperidine and the amide group. When fragmentation occurred at this site, opposite lower fragments at *m*/*z* 206.0616 for 4F-FUF and 164.1071 for iBF were observed. In addition, a minor fragment at *m*/*z* 134.0965 was observable for both compounds and originated from the phenylethyl moiety attached to the methylamine residue of the piperidine ring after cleavage. Cleavage was possible also in different points of the piperidine ring, leading to the fragments *m*/*z* 160.1124 and 272.1085 for 4F-FUF and 230.1542 and 204.1386 for iBF. For 4-FFUF, the unchanged piperidine ring led to fragment *m*/*z* 84.0813, whereas the fragment at *m*/*z* 281.2017 corresponded to the elimination of isobutyraldehyde through amide cleavage for iBF.

#### 2.3.1. 4F-FUF Metabolites

All the spectra with the postulated fragmentation are reported in Figure S3. The presence of the main fragments *m*/*z* 188.1435 and 105.0703 in the metabolite spectra suggested that the phenethylpiperidine structure was unchanged. This was the case for M19_FFUF, which was produced by amide hydrolysis of 4F-FUF and for the most abundant metabolite, M14_FFUF, which was putatively annotated as a dihydrodiol metabolite of 4-FFUF. Based on the fragment 299.1925, it can be hypothesized that the dihydrodiol formation site is the furan ring; even if this structure was postulated on a minor fragment, it must be highlighted that based on the literature data, this was the most probable structure [19]. Based on the fragmentation pattern, the phenetylpiperidine moiety was unchanged also for M2_FFUF and M9_FFUF, which were phase II glucuronide conjugates. In M2_FFUF the glucuronic acid was linked to the aniline ring in the para position subsequently to oxidative defluorination, whereas conjugation occurred at the opened furanyl ring in M9_FFUF. In both metabolites a fragment arising from the loss of the glucuronic acid moiety (loss of 176.0317 u) was observed, reinforcing the hypothesis that they were glucuronides. The fragments *m*/*z* 188.1435 and 105.0703 were found also in the spectra of the metabolites arising from furanyl ring scission and subsequent oxidation (M12_FFUF) and taurine conjugation (M5_FFUF) or hydroxylation (M8_FFUF and M13_FFUF) with no biotransformations on the other sites of the molecule. An analogue defluorinated metabolite of M12_FFUF was identified by Watanabe et al. in their work on furanylfentanyl metabolites. For these metabolites the fragment *m*/*z* 299.1919, which arose from amide cleavage, was observed in the spectra; this fragmentation pathway was favored by the formation of an α,β-unsaturated carbonyl system consequently to furan ring opening and was rarely observed in the other metabolites and in the parent compound. M15_FFUF is an isomer of M12_FFUF—due to the absence of the fragment 299, we suppose that for this metabolite the furan ring was unopened. Concerning M5_FFUF, to the best of our knowledge this is the first report of the formation of a taurine-conjugated metabolite for fentanyl analogues; the presence of the fragment 409.1918, which corresponded to the loss of the taurine moiety (C_2_H_7_NO_3_S) supported the hypothesized structure.

When *N*-dealkylation occurred (M4_FFUF), the typical phenethylpiperidine fragments were obviously not observed; *m*/*z* 84.0815, which corresponded to the elimination of the piperidine, was the base peak. A dihydrodiol *N*-dealkylated metabolite was also detected (M3_FFUF). The spectrum showed minor fragments, such as *m*/*z* 101.0238 (C_4_H_5_O_3_), which corresponded to the dihydrodiolfuranyl moiety, suggesting that dihydrodiol formation occurred on the furanyl ring.

Hydroxylation at the phenethylpiperidine moiety was observed for a number of metabolites. This transformation was indicated by the shift of fragment *m*/*z* 188.1435 and/or 105.0703 by 16 u. For M6_FFUF, M7_FFUF, M16_FFUF and M17_FFUF, the hydroxylation occurred on the phenylethyl moiety, confirmed by the presence of both *m*/*z* 204.1384 and 121.0650, whereas for M10_FFUF, M11_FFUF and M18_FFUF, hydroxylation at the piperidine ring was demonstrated by the phenethyl moiety being left unchanged (the presence of the fragment *m*/*z* 105.0703) and the fragments 204.1384 and 186.1278 resulting from H_2_O elimination from the former fragment. In the spectrum of M10_FFUF a fragment at *m*/*z* 188 was also detected; a likely explanation is that there was the interference of another isomer which was not chromatographically resolved, so that the postulated structure of this metabolite is ambiguous.

#### 2.3.2. iBF Metabolites

All the spectra with the postulated fragmentation are reported in Figure S4.

The same observations made for 4F-FUF can be exploited to elucidate iBF metabolite structures. The unchanged phenetylpiperidine moiety was testified by the presence of the already discussed fragments 188.1435 and 105.0703; these fragments were both detected in the MS/MS spectra of several metabolites, showing that several biotransformations occurred on the isobutyryl moiety or possibly on the aniline ring. The examination of minor fragments in the spectra generally served to determine the exact position of the modifications; for example, for M15_iBF the fragment at *m*/*z* 85.0290, corresponding to C_4_H_5_O_2_, suggested the elimination of a hydroxylated isobutyryl moiety. Similarly, for M16_iBF, the fragment *m*/*z* 337.2278 resulted from elimination of a CH_2_O group from the isobutyryl region. Regarding M17_iBF, M18_iBF and M7_iBF, the presence of the fragment 281.2013 indicated that the aniline group was not substituted; thus, the isobutyryl moiety should carry the carboxy, hydroxy and glucuronide groups, respectively. M4_iBF, with *m*/*z* 206.1542, which was identical to the 4-FFUF metabolite M1_FFUF, arose from the addition of an -OH to the phenetylpiperidine moiety. This metabolite was produced by an oxidative *N*-dealkylation reaction, so that the hydroxyl group substituted the amide nitrogen in position 4 of the piperidine ring.

For M8_iBF, no minor fragments indicated the position of the glucuronide conjugation; however, being a phase II metabolite, it probably derives from M16_iBF or M18_iBF, which were putatively annotated as aliphatic hydroxylated metabolites. On the other hand, M12_iBF was an isomer of M8_iBF, with the glucuronide on the aromatic ring of the phenethylpiperidine, indicated by the presence of the fragments *m*/*z* 204.1384 and 121.0650. These two fragments were also found in the spectra of M6_iBF and showed that a hydroxylation occurred on the aromatic ring, whereas the aliphatic carboxylation was supported by the fragments 353.2219 (−CO_2_) and 297.1960, obtained through the elimination of the carboxylated isobutyryl. The isomer M11_iBF had a similar spectrum, but the absence of the fragment 121.0650 suggested that the hydroxylation occurred on the piperidine ring. Hydroxylation at the piperidine ring was hypothesized for other metabolites, including the hydroxylated M20_iBF, the dihydroxylated M9_iBF, M19_iBF and the glucuronide conjugate M5_iBF; however, for all these metabolites the exact position of the -OH groups could not be deduced. For all these metabolites the fragment 186.1280, resulting from hydroxylation followed by H_2_O elimination, was detected. Likewise, M22_iBF, which was putatively annotated as a *N*-oxide metabolite of iBF, also on the basis of the high Rt, showed this fragment. In the spectra of M14_iBF and M21_iBF, the fragment 202.1229 was detected instead of the 204, showing that oxidation to carbonyl metabolites occurred at the piperidine ring and the ethyl, respectively. For the dihydroxylated metabolites, the position of second hydroxylation was the isobutyryl moiety, based on the fragments 279.1855 in the spectrum of M9_iBF and 297.1960 for M19_iBF, which suggested that only one hydroxylation occurred on the phenylethylpiperidine ring and that the aromatic group was also unchanged. Finally, for metabolite M13_iBFm aromatic dihydroxylation followed by methylation and glucuronide conjugation was hypothesized based on the presence of the fragment *m*/*z* 151.0755; this fragment differed from fragment 105.0703, which corresponds to the unchanged phenethyl moiety, by 46 u, suggesting that the biotransformation site was the aromatic moiety. For all glucuronides, the typical loss of glucuronic acid (*m*/*z* 176) was observed.

Concerning the metabolites of iBF which were *N*-dealkylated, similarly to 4F-FUF metabolites, a main peak at *m*/*z* 84.0815, which corresponded to the piperazine ring, was detected. M10_iBF was putatively identified as the nor-iBF metabolite, which arose from *N*-dealkylation of P_iBF; M2_iBF originated from M10_iBF by hydroxylation and was putatively identified on the basis of fragment *m*/*z* 177.1388, which suggested that the site of the biotransformation was the isobutyryl moiety. M1_iBF was recognized as the corresponding phase II of M2_iBF, which was formed after glucuronidation of the hydroxy group; similarities in the spectra and the typical shift of *m/z* 176 were observed. For M3_iBF, the position of the carboxylic acid could not be deduced from the spectra, but once again carboxylation was only possible on the isobutyryl ring.

## 3. Materials and Methods

### 3.1. Chemicals and Reagents

4F-FUF, iBF, diclofenac (sodium salt) and testosterone were purchased from Cayman Chemicals (Ann Arbor, MI, USA). Pooled cryopreserved male mouse hepatocytes, Williams’ E Medium (phenol red free), cell maintenance supplement pack (dexamethasone, cocktail B (penicillin-streptomycin, (insulin, transferrin, selenium complex, (ITS) bovine serum albumine (BSA) and linoleic acid), GlutaMAX™ and HEPES), as well as a thawing and plating supplement pack containing prequalified fetal bovine serum (FBS), dexamethasone, Cocktail A (FBS, penicillin, streptomycin human recombinant insulin, GlutaMAX™ and HEPES), were purchased from Life Technologies (Monza, Italy). Formic acid, methanol, acetonitrile and water were obtained from Fisher Scientific (Fair Lawn, NJ, USA). All solvents employed in the incubation and chromatographic system were ultra-performance liquid chromatography (UHPLC) grade.

Ethanol (BioUltra, for molecular biology, ≥99.8%) and TWEEN^®^ 80 for the in vivo study were purchased from Sigma-Aldrich, whereas physiological solution (0.9% *v*/*v* NaCl) was obtained from Eurospital, S.p.A, (Trieste TS, Italy).

### 3.2. In Vitro Incubation Using Mouse Hepatocytes

For in vitro experiments, both NPSs, dissolved in acetonitrile, were incubated at 5 μmol L^−1^ and 37 °C with mouse cryopreserved hepatocytes. Cells were thawed in and washed with Williams’ E Medium, containing dexamethasone (1 μmol L^−1^), and cocktail A, which contained penicillin/streptomycin (1%), human recombinant insulin (4 μg mL^−1^), Glutamax™ (2 mmol L^−1^), HEPES pH 7.4 (15 mmol L^−1^) and FBS (5%) and centrifuged at 55× *g* for 3 min at room temperature. After centrifugation and removal of the supernatant, the cell pellet was resuspended in Williams’ E Medium, containing dexamethasone (0.1 μmol L^−1^) and cocktail B, containing penicillin/streptomycin (0.5%), human recombinant insulin (6.25 μg mL^−1^), human transferrin (6.25 μg mL^−1^), selenous acid (6.25 ng mL^−1^), BSA 1.25 mg mL^−1^, linoleic acid (5.35 μg mL^−1^), Glutamax™ (2 mmol L^−1^) and HEPES pH 7.4 (15 mmol L^−1^). Cell viability was assessed with the Trypan blue 0.4% exclusion method. 4F-FUF and iBF molecules were incubated in duplicate in 800 μL of a 1.10^6^ cell mL^−1^ suspension at 37 °C in a water bath under constant gentle shaking. Diclofenac and testosterone were also incubated, as a positive control, to verify metabolic capability under our experimental conditions: diclofenac was used as positive control for phase I metabolism and testosterone was used for phase II metabolism. Negative controls, i.e., hepatocytes without drugs and drugs without hepatocytes, were also included in the experimental study. 200-μL sample aliquots were collected at 0.5, 1, 2 and 3 h; reaction quenching was obtained by the addition of 200 μL acetonitrile. Specimens were stored at −20 °C until analysis. Before injection, samples were centrifuged; the supernatant was removed, diluted 1:4 with water and filtered with Minisart SRP25 4 mm (0.45 μm) syringe filters (Sartorius, Turin, Italy).

### 3.3. In Vivo Study on Mice

Sixteen-male ICR (CD-1^®^) mice weighing 30–35 g (Centralized Preclinical Research Laboratory, University of Ferrara, Italy) were group-housed (5 mice per cage; floor area per animal was 80 cm^2^; minimum enclosure height was 12 cm), exposed to a 12:12-h light-dark cycle (light period from 6:30 AM to 6:30 PM) at a temperature of 20 °C–22 °C and humidity of 45–55% and were provided with ad libitum access to food (Diet 4RF25 GLP; Mucedola, Settimo Milanese, Milan, Italy) and water. The experimental protocols performed in the present study were in accordance with the U.K. Animals (Scientific Procedures) Act of 1986 and associated guidelines and the new European Communities Council Directive of September 2010 (2010/63/EU). Experimental protocols were approved by the Italian Ministry of Health (license No. 335/2016-PR) and by the Animal Welfare Body of the University of Ferrara. According to the ARRIVE guidelines, all possible efforts were made to minimize the number of animals used, to minimize the animals’ pain and discomfort and to reduce the number of experimental subjects. For the overall study, 16 mice were used. In the analysis of urine excretion studies for vehicle (blank control) 4 mice were used, whereas for each treatment (4F-FUF and iBF both at 5 mg/kg) 6 mice were used (total: 12).

For the studies, mice were administered with 4F-FUF or iBF dissolved in absolute ethanol (final concentration of 2% *v*/*v*) and Tween 80 (2% *v*/*v*) and brought to its final volume with saline (0.9% NaCl *v*/*v*). The solution made with ethanol, Tween 80 and saline was also used as the vehicle (blank control). The drugs were administered by intraperitoneal injection at a volume of 4 μL/g; the final concentration of 4F-FUF or iBF was 5 mg/kg. The control group of 4 mice was administered only with vehicle solution. The mice were single-housed (one mouse per metabolic cage, with free access to food and water) in a colony room under constant temperature (23 °C–24 °C) and humidity (45–55%). Urine samples were collected in 2-mL tubes before drug injections (control), and every hour for 6 consecutive hours from the administration of the treatments [9,33] After 6 h, urine was collected cumulatively in the 6–12, 12–24 and 24–36 h time interval and stored at −20 °C until analysis.

Before LC-HRMS analysis, urine samples were diluted 1:4 with water and filtered with Minisart SRP25 4 mm (0.45 μm) syringe filters (Sartorius, Turin, Italy).

### 3.4. LC-HRMS Analysis

A Thermo Scientific Ultimate 3000 RSLC system coupled with a Thermo Scientific Q-Exactive Mass spectrometer (Thermo Fisher Scientific, Bremen, Germany) was used for analysis.

Chromatographic separation was carried out with an Excel 2 C18-PFP (100 × 2.1 mm ID) column from Ace (Aberdeen, Scotland) packed with particles of 2 μm, maintained at 35 °C at a flow rate of 0.5 mL min^−1^.

Mobile phases consisted of 0.1% (*v*/*v*) formic acid + 10 mM ammonium formate in water (Phase A) and 0.1% formic acid in acetonitrile (Phase B). The gradient started with 0% B and these conditions were maintained for one min; phase B was then increased to 25% in two min, to 35% in the following two min and held for three min. Phase B was then ramped to 50% over 1.5 min and to 100% in 0.5 min; it was kept stable for one min and then equilibrated to the initial conditions, yielding a total runtime of 12.5 min. Injection volume was 6 μL.

The Q-Exactive mass spectrometer was equipped with a heated electrospray ionization source (HESI-II) operated in positive mode; mass spectra were acquired in full scan/data dependent in the range 50–800 *m*/*z*. The operating parameters of the ion source were set as follows: spray voltage 3.5 kV, capillary temperature 350 °C, heater temperature 300 °C, S-lens RF level 60, sheath gas flow rate 55, auxiliary gas flow rate 20. Nitrogen was used for spray stabilization, for collision-induced dissociation experiments in the high energy collision dissociation (HCD) cell and as the damping gas in the C-trap.

The instrument was calibrated in the positive and negative mode every working day. For full scan, resolution was 70,000 (FWHM at *m*/*z* 200), whereas automatic gain control (AGC) and maximum injection time were set at 1 × 10^5^ and 100 ms, respectively. In MS/MS mode, resolution was 35,000 (FWHM at *m*/*z* 200) and three different collision energies, i.e., 10, 30, 50, were applied.

### 3.5. Data Analysis

The raw files obtained from the in vitro and in vivo studies were processed separately using Compound Discoverer™ 2.0 (Thermo Scientific™, Waltham, MA, USA) with a specific workflow for metabolite identification which encompassed all the common biotransformation reactions, as well as untargeted nodes. For each study an output table including *m*/*z* versus retention time versus raw peak intensity for all the analyzed samples was generated. Potential metabolites detected in the negative control, 0 h samples or in the degradation controls were excluded. The different features were evaluated individually and only compounds with a reasonable elemental composition (1 < *N* < 3; C < 30; 0 < 10), an acceptable peak shape and area above 10,000 were considered as potential metabolites. MS/MS fragmentation spectra associated with the precursor ions were then evaluated for structure annotation. Given that no standards were available, only putative identification was possible [34].

## 4. Conclusions

The metabolic profiles of iBF and 4F-FUF were investigated in this study. For the first compound the *N*-dealkylated metabolite (nor-isobutyrylfentanyl) was the relatively most intense but hydroxylation and subsequent carbonylation of the parent compound was also a main transformation, leading to two different isomers; all these metabolites can be considered good biomarkers for iBF consumption in biological samples. For 4F-FUF, the main metabolite was the dihydrodiol derivative, which was further *N*-dealkylated to produce the second most relatively intense metabolites in vivo, whereas *N*-dealkylation of the parent compound prevailed in vitro. Despite these differences, in general there was a good agreement between in vitro and in vivo samples; in fact, the main metabolites were found in both studies, confirming that hepatocyte incubation is a good approach for metabolite profiling and for the identification of suitable biomarkers for analytical methods. However, it must be taken into account that the relative abundance of metabolites may be different in authentic biological samples.

A limitation of our in vivo study was that it was based on an animal model and no real samples from human consumers were analyzed. On the other hand, controlled administration of drugs to animals has the advantage of providing several samples at different time points to obtain pharmacokinetic parameters analogously to preclinical studies. Analysis of real human samples is desirable; however, in NPS metabolism studies these are often collected from autopsies and scarce or no information about dosage and time of intake is provided. In these cases, post-mortem redistribution may be responsible for unclear results.

## Figures and Tables

**Table 1:** List of proposed metabolites of 4-fluoro-furanylfentanyl with the main identification parameters and postulated biotransformation. The relatively most intense metabolites are in bold. Rt—retention time.

ID	Biotransformation	Rt (min)	Formula	Measured *m*/*z*	Mass Error (PPM)	Diagnostic Ions
						-
M1_FFUF	Oxidative *N*-dealkylation	4.05	C_13_H_20_NO	206.1547	1.02	188.1433, 105.0702, 56.0603
M2_FFUF	Amide hydrolysis + oxidative defluorination + glucuronidation	4.08	C_25_H_33_N_2_O_7_	473.2289	0.26	188.1433, 297.1960, 105.0702
**M3_FFUF**	**Dihydrodiol formation + *N*-dealkylation**	**4.10**	**C_16_H_20_FN_2_O_4_**	**323.1404**	**−0.96**	**84.0814, 194.0810, 166.0863**
M4_FFUF	Oxidative *N*-dealkylation	5.29	C_16_H_18_FN_2_O_2_	289.1346	−2.18	84.0814, 206.0611, 56.0503
M5_FFUF	Oxidation (furanyl ring opened) + oxidation to carbonyl metabolite + taurine conjugation	5.35	C_26_H_33_FN_3_O_6_S	534.2069	−0.95	188.1433, 105.0703, 299.1917, 409.1917
M6_FFUF	Dihydrodiol formation + hydroxylation	5.37	C_24_H_28_FN_2_O_5_	443.1973	−2.09	121.0650, 204.1382, 323.1401
M7_FFUF	Dihydrodiol formation + hydroxylation	5.64	C_24_H_28_FN_2_O_5_	443.1969	−2.99	204.1382, 121.0650, 335.1400
M8_FFUF	Oxidation (furanyl ring opened) + reduction	5.65	C_24_H_32_FN_2_O_3_	415.2384	−3.12	188.0702, 105.0702, 299.1918
M9_FFUF	Oxidation (furanyl ring opened + glucuronidation)	5.67	C_30_H_39_FN_2_O_9_	589.2552	−1.59	188.1433, 105.0702, 413.2233, 299.1917
M10_FFUF	Oxidation (furanyl ring opened) + hydroxylation	5.80	C_24_H_30_FN_2_O_4_	429.2186	−0.84	186.1277, 204.1386, 299.1918
M11_FFUF	Dihydrodiol formation + hydroxylation	5.90	C_24_H_28_FN_2_O_5_	443.1976	−1.41	425.1868, 186.1277, 134.0965
M12_FFUF	Oxidation (furanyl ring opened)	6.50	C_24_H_28_FN_2_O_3_	411.2078	−1.45	188.1434, 105.0703, 299.1921
M13_FFUF	Oxidation (furanyl ring opened)	6.60	C_24_H_30_FN_2_O_3_	413.2237	−0.84	188.1434, 105.0703, 299.1916
**M14_FFUF**	**Dihydrodiol formation**	**6.77**	**C_24_H_28_FN_2_O_4_**	**427.2027**	**−1.43**	**188.1434, 105.0703, 335.1401**
M15_FFUF	Hydration	6.92	C_24_H_28_FN_2_O_3_	411.2085	0.25	188.1434, 105.0703
M16_FFUF	Hydroxylation	7.02	C_24_H_26_FN_2_O_3_	409.1915	−3.04	204.1382, 121.0650
M17_FFUF	Hydroxylation	7.35	C_24_H_26_FN_2_O_3_	409.1915	−3.04	204.1382, 121.0650
M18_FFUF	Hydroxylation	7.87	C_24_H_26_FN_2_O_3_	409.1912	−3.78	204.1385, 186.1278, 391.1817
M19_FFUF	Amide hydrolysis	9.43	C_19_H_24_FN_2_	299.1918	−1.84	105.0703, 204.1385, 186.1277
P_FFUF	-	9.62	C_24_H_26_FN_2_O_2_	393.1974	−1.10	-
M20_FFUF	*N*-oxygenation	10.58	C_24_H_26_FN_2_O_3_	409.1912	−3.78	186.1277, 204.1386, 349.2273

**Table 2:** Results of the in vitro and in vivo studies for 4-FUF.

ID	Average (*n* = 2) Peak Area in Hepatocyte Samples				Average Area in Urine Samples (CV%)								
	0.5 h	1 h	2 h	3 h	1 h	2 h	3 h	4 h	5 h	6 h	6–12 h (*n* = 6)	12–24 h (*n* = 5)	24–31 h (*n* = 5)
M1_FFUF	4.35 × 10^6^ (13)	5.86 × 10^6^ (6)	5.52 × 10^6^ (9)	2.30 × 10^6^ (11)	NS	NS	NS	NS	NS	NS	1.08 × 10^7^ (51)	1.15 × 10^5^ (67)	2.28 × 10^5^ (63)
M2_FFUF	2.77 × 10^6^ (11)	1.09 × 10^7^ (8)	2.21 × 10^7^ (16)	3.39 × 10^6^ (6)	NS	NS	NS	NS	NS	NS	6.96 × 10^8^ (50)	4.71 × 10^7^ (44)	5.54 × 10^7^ (60)
M3_FFUF	1.58 × 10^6^ (5)	5.42 × 10^6^ (9)	7.49 × 10^6^ (12)	2.62 × 10^6^ (10)	NS	NS	NS	NS	NS	NS	5.55 × 10^9^ (42)	2.29 × 10^8^ (40)	2.83 × 10^8^ (77)
M4_FFUF	9.34 × 10^7^ (11)	1.66 × 10^8^ (16)	1.66 × 10^8^ (13)	7.12 × 10^7^ (8)	NS	NS	NS	NS	NS	NS	3.91 × 10^8^ (47)	1.79 × 10^7^ (38)	1.82 × 10^7^ (54)
M5_FFUF	NF	7.53 × 10^4^ (18)	1.1 × 10^5^ (11)	NF	NS	NS	NS	NS	NS	NS	7.89 × 10^7^ (51)	6.6 × 10^6^ (44)	6.32 × 10^6^ (64)
M6_FFUF	NF	NF	NF	NF	NS	NS	NS	NS	NS	NS	1.96 × 10^9^ (32)	2.27 × 10^8^ (39)	3.21 × 10^8^ (56)
M7_FFUF	NF	NF	NF	NF	NS	NS	NS	NS	NS	NS	1.01 × 10^8^ (29)	1.05 × 10^7^ (44)	1.84 × 10^7^ (32)
M8_FFUF	5.63 × 10^6^ (9)	1.07 × 10^7^ (11)	9.98 × 10^6^ (11)	5.62 × 10^6^ (13)	NS	NS	NS	NS	NS	NS	3.05 × 10^7^ (50)	1.26 × 10^6^ (14)	1.37 × 10^6^ (56)
M9_FFUF	2.94 × 10^5^ (8)	7.74 × 10^5^ (13)	1.16 × 10^5^ (14)	2.03E5 (11)	NS	NS	NS	NS	NS	NS	3.81 × 10^7^ (66)	4.89 × 10^6^ (55)	3.11 × 10^6^ (49)
M10_FFUF	3.08 × 10^6^ (15)	6.57 × 10^6^ (12)	5.33 × 10^6^ (7)	4.52 × 10^6^ (16)	NS	NS	NS	NS	NS	NS	5.66 × 10^8^ (63)	3.94 × 10^7^ (43)	5.70 × 10^7^ (55)
M11_FFUF	3.47 × 10^6^ (6)	8.55 × 10^6^ (15)	1.01 × 10^7^ (18)	3.17 × 10^6^ (18)	NS	NS	NS	NS	NS	NS	7.06 × 10^8^ (44)	9.71 × 10^7^ (21)	2.23 × 10^8^ (41)
M12_FFUF	6.09 × 10^6^ (9)	1.28 × 10^7^ (17)	3.35 × 10^6^ (11)	2.56 × 10^6^ (15)	NS	NS	NS	NS	NS	NS	9.22 × 10^6^ (73)	1.26 × 10^4^ (51)	5.66 × 10^4^ (62)
M13_FFUF	8.02 × 10^7^ (10)	1.4 × 10^8^ (9)	6.60 × 10^7^ (11)	7.67 × 10^7^ (20)	NS	NS	NS	NS	NS	NS	1.55 × 10^8^ (69)	2.04 × 10^6^ (31)	2.44 × 10^6^ (55)
M14_FFUF	3.59 × 10^8^ (4)	6.33 × 10^8^ (18)	7.15 × 10^8^ (12)	2.57 × 10^8^ (12)	NS	NS	NS	NS	NS	NS	1.77 × 10^10^ (55)	2.85 × 10^9^ (19)	3.33 × 10^9^ (50)
M15_FFUF	NF	NF	NF	NF	NS	NS	NS	NS	NS	NS	8.11 × 10^6^ (47)	9.88 × 10^5^ (33)	6.44 × 10^5^ (49)
M16_FFUF	5.11 × 10^5^ (16)	1.11 × 10^6^ (5)	4.31 × 10^6^ (7)	8.31 × 10^5^ (17)	NS	NS	NS	NS	NS	NS	8.26 × 10^6^ (75)	3.23 × 10^6^ (59)	1.51 × 10^6^ (72)
M17_FFUF	NF	NF	NF	NF	NS	NS	NS	NS	NS	NS	2.91 × 10^6^ (59)	NF	NF
M18_FFUF	6.00 × 10^6^ (18)	1.20 × 10^7^ (9)	5.75 × 10^6^ (14)	7.57 × 10^6^ (14)	NS	NS	NS	NS	NS	NS	3.27 × 10^6^ (61)	NF	NF
M19_FFUF	7.87 × 10^6^ (11)	4.19 × 10^7^ (16)	3.78 × 10^7^ (7)	5.02 × 10^7^ (9)	NS	NS	NS	NS	NS	NS	3.40 × 10^8^ (32)	1.69 × 10^7^ (44)	2.09 × 10^7^ (19)
P_FFUF	4.05 × 10^8^ (12)	7.47 × 10^8^ (6)	2.83 × 10^8^ (9)	3.91 × 10^8^ (13)	NS	NS	NS	NS	NS	NS	2.60 × 10^9^ (69)	5.95 × 10^7^ (71)	8.62 × 10^7^ (61)
M20_FFUF	1.2 × 10^6^ (8)	8.86 × 10^6^ (19)	5.63 × 10^6^ (21)	1.02 × 10^7^ (12)	NS	NS	NS	NS	NS	NS	5.60 × 10^6^ (81)	4.53 × 10^6^ (79)	1.98 × 10^6^ (85)

NS no sample available, NF not found.

**Table 3:** List of proposed metabolites of isobutyrylfentanyl with the main identification parameters and postulated biotransformation. The relatively most intense metabolites are in bold.

ID	Biotransformation	Rt (min)	Formula	*m*/*z*	Mass Error (PPM)	Diagnostic Ions
						-
M1_iBF	*N*-dealkylation + hydroxylation + gluconidation	3.61	C_21_H_31_N_2_O_8_	439.2088	1.73	263.1753, 84.0814, 180.1019
**M2_iBF**	***N*-dealkylation + hydroxylation**	**3.86**	**C_15_H_23_N_2_O_2_**	**263.1758**	**−0.58**	84.0814, 245.1648, 177.1387
M3_iBF	*N*-dealkylation + oxidation	3.89	C_15_H_21_N_2_O_3_	277.1546	−2.23	84.0814, 233.1648
M4_iBF	Oxidative *N*-dealkylation	4.05	C_13_H_20_NO	206.1542	−1.40	188.1433, 105.0702
M5_iBF	Dihydroxylation + glucuronidation	4.78	C_29_H_39_N_2_O_9_	559.2642	−2.42	263.1390, 383.2328, 204.1382, 116.0709
M6_iBF	Oxidation + hydroxylation	4.94	C_23_H_29_N_2_O_4_	397.2119	−2.10	204.1384, 121.0651, 353.2221
M7_iBF	Oxidation to carbonyl metabolite + glucuronidation	5.34	C_29_H_37_N_2_O_9_	557.2490	−1.63	188.1434, 337.2272, 105.0703
M8_iBF	Hydroxylation + glucuronidation	5.37	C_29_H_39_N_2_O_8_	543.2706	−0.08	188.1433, 367.2378, 105.0702
M9_iBF	Dihydroxylation	5.37	C_23_H_31_N_2_O_3_	383.2330	−1.22	204.1384, 186.1278, 365.2223
**M10_iBF**	**Oxidative *N*-dealkylation**	**5.39**	**C_15_H_23_N_2_O**	**247.1808**	**−0.96**	84.0813, 177.1386, 164.1073
M11_iBF	Oxidation to carbonyl metabolite+ hydroxylation	5.45	C_23_H_29_N_2_O_4_	397.2119	−2.10	204.1384, 353.2222, 121.0651
M12_iBF	Hydroxylation +glucuronidation	5.56	C_29_H_39_N_2_O_8_	543.2707	0.11	367.2376, 204.1382, 121.0650
M13_iBF	Dihydroxylation + methylation + glucuronidation	5.64	C_30_H_41_N_2_O_9_	573.2809	−0.53	397.2534, 410.1813, 234.1488
**M14_iBF**	**Oxidation + hydroxylation**	**5.77**	**C_23_H_29_N_2_O_3_**	**381.2181**	**0.74**	202.1229, 148.0759, 105.0703
M15_iBF	Hydroxylation	5.87	C_23_H_29_N_2_O_2_	365.2225	−1.10	188.1436, 105.0704, 244.1332
M16_iBF	Hydroxylation	6.09	C_23_H_31_N_2_O_2_	367.2381	−1.23	188.1434, 105.0703, 246.1486
**M17_iBF**	**Oxidation to carbonyl metabolite**	**6.16**	**C_23_H_29_N_2_O_3_**	**381.2190**	**3.10**	188.1434, 105.0703, 281.2011, 337.2272
M18_iBF	Hydroxylation	6.43	C_23_H_31_N_2_O_2_	367.2381	−1.23	188.1434, 105.0703, 281.2013
M19_iBF	Dihydroxylation	7.81	C_23_H_31_N_2_O_3_	383.2342	1.91	105.0703, 186.1274, 275.1753
M20_iBF	Hydroxylation	7.84	C_23_H_31_N_2_O_2_	367.2381	−1.23	186.1274, 204.1386, 105.0703
M21_iBF	Oxidation	8.80	C_23_H_29_N_2_O_2_	365.2226	−0.83	202.1230, 195.1808, 230.1536
P_iBF	-	9.61	C_23_H_31_N_2_O	351.2428	−2.39	-
M22_iBF	*N*-oxidation	10.54	C_23_H_29_N_2_O_2_	367.2381	−1.23	186.1274, 105.0703, 204.1386

**Table 4:** Results of the in vitro and in vivo studies for isobutyrylfentanyl.

ID	Average (*n* = 2) Peak Area in Hepatocyte Samples (CV%)				Average Area in Urine Samples (CV%)								
	0.5 h	1 h	2 h	3 h	1 h (*n* = 1)	2 h (*n* = 2)	3 h (*n* = 6)	4 h	5 h (*n* = 6)	6 h (*n* = 5)	6–12 h (*n* = 6)	12–24 h (*n* = 6)	24–31 h (*n* = 6)
M1_iBF	NF	NF	NF	NF	4.08 × 10^7^	1.53 × 10^8^ (17)	9.85 × 10^8^ (74)	NS	2.65 × 10^8^ (50)	1.51 × 10^8^ (51)	8.41 × 10^7^ (63)	1.94 × 10^7^ (53)	2.86 × 10^7^ (52)
M2_iBF	1.49 × 10^7^ (5)	1.64 × 10^7^ (9)	3.60 × 10^7^ (8)	2.00 × 10^7^ (10)	8.09 × 10^8^	2.01 × 10^9^ (32)	3.31 × 10^9^ (51)	NS	2.76 × 10^8^ (49)	2.06 × 10^8^ (50)	1.14 × 10^9^ (31)	3.55 × 10^8^ (33)	4.26 × 10^8^ (55)
M3_iBF	NF	NF	NF	NF	3.58 × 10^7^	5.58 × 10^7^ (15)	2.03 × 10^8^ (39)	NS	1.93 × 10^8^ (46)	1.20 × 10^8^ (49)	7.93 × 10^7^ (29)	1.51 × 10^7^ (43)	2.01 × 10^7^ (58)
M4_iBF	2.87 × 10^7^ (18)	1.86 × 10^7^ (10)	3.88 × 10^7^ (8)	1.76 × 10^7^ (12)	9.98 × 10^7^	3.37 × 10^7^ (26)	1.88 × 10^8^ (71)	NS	6.59 × 10^7^ (33)	2.80 × 10^7^ (39)	1.94 × 10^7^ (61)	3.54 × 10^6^ (44)	4.88 × 10^6^ (62)
M5_iBF	NF	NF	NF	NF	5.55 × 10^7^	3.49 × 10^7^ (32)	1.43 × 10^8^ (49)	NS	9.10 × 10^7^ (39)	8.16 × 10^7^ (61)	7.42 × 10^7^ (62)	1.24 × 10^7^ (38)	2.63 × 10^7^ (43)
M6_iBF	NF	NF	NF	NF	3.44 × 10^7^	1.21 × 10^7^ (18)	1.03 × 10^8^ (52)	NS	4.97 × 10^7^ (40)	3.36 × 10^7^ (59)	2.48 × 10^7^ (36)	9.57 × 10^6^ (52)	1.81 × 10^7^ (43)
M7_iBF	NF	NF	NF	NF	1.18 × 10^8^	3.91 × 10^7^ (19)	2.41 × 10^8^ (50)	NS	2.49 × 10^8^ (33)	2.26 × 10^8^ (39)	1.51 × 10^8^ (44)	2.24 × 10^7^ (42)	4.14 × 10^7^ (51)
M8_iBF	2.76 × 10^5^ (19)	1.39 × 10^6^ (11)	1.45 × 10^6^ (9)	7.50 × 105 (11)	1.28 × 10^8^	6.02 × 10^7^ (21)	1.54 × 10^8^ (38)	NS	1.50 × 10^8^ (52)	2.75 × 10^8^ (44)	1.66 × 10^8^ (32)	3.70 × 10^7^ (41)	4.94 × 10^7^ (54)
M9_iBF	1.51 × 10^7^ (7)	1.65 × 10^7^ (9)	2.12 × 10^7^ (6)	1.41 × 10^7^ (18)	4.69 × 10^6^	1.60 × 10^7^ (32)	4.15 × 10^7^ (30)	NS	5.29 × 10^7^ (53)	3.59 × 10^7^ (41)	3.66 × 10^7^ (61)	3.45 × 10^6^ (59)	5.55 × 10^6^ (37)
M10_iBF	2.04 × 10^9^ (8)	2.01 × 10^9^ (6)	2.68 × 10^9^ (8)	1.62 × 10^9^ (5)	6.78 × 10^9^	1.21 × 10^10^ (18)	1.40 × 10^10^ (29)	NS	1.08 × 10^10^ (50)	1.16 × 10^10^ (39)	6.96 × 109 (51)	2.35 × 10^9^ (41)	2.83 × 10^9^ (38)
M11_iBF	NF	NF	NF	NF	1.27 × 10^8^	1.74 × 10^7^ (44)	2.12 × 10^8^ (61)	NS	1.88 × 10^8^ (48)	1.18 × 10^8^ (41)	1.27 × 10^8^ (52)	2.86 × 10^7^ (32)	5.49 × 10^7^ (34)
M12_iBF	1.59 × 10^6^ (6)	1.61 × 10^6^ (5)	3.47 × 10^6^ (6)	1.45 × 10^6^ (7)	7.01 × 10^7^	5.06 × 10^7^ (32)	1.05 × 10^8^ (50)	NS	9.86 × 10^7^ (33)	1.53 × 10^8^ (41)	1.19 × 10^8^ (54)	3.70 × 10^7^ (61)	4.84 × 10^7^ (39)
M13_iBF	NF	NF	NF	NF	9.79 × 10^5^	1.62 × 10^6^ (11)	3.54 × 10^6^ (41)	NS	4.44 × 10^6^ (39)	1.47 × 10^7^ (39)	3.41 × 10^6^ (41)	8.66 × 10^5^ (34)	8.55 × 10^5^ (31)
M14_iBF	1.73 × 10^6^ (6)	2.00 × 10^6^ (9)	2.55 × 10^6^ (10)	1.70 × 10^6^ (12)	1.38 × 10^9^	5.46 × 10^8^ (36)	3.40 × 10^9^ (38)	NS	2.61 × 10^9^ (41)	2.15 × 10^9^ (49)	1.51 × 10^9^ (61)	3.93 × 10^8^ (36)	6.15 × 10^8^ (18)
M15_iBF	6.61 × 10^6^ (9)	3.98 × 10^5^ (8)	6.63 × 10^6^ (9)	7.50 × 10^6^ (7)	4.84 × 10^5^	8.69 × 10^5^ (18)	1.45 × 10^6^ (42)	NS	6.20 × 10^5^ (52)	1.21 × 10^6^ (51)	2.93 × 10^5^ (55)	4.49 × 104 (31)	2.30 × 104 (54)
M16_iBF	2.71 × 10^8^ (8)	2.52 × 10^8^ (10)	2.74 × 10^8^ (8)	1.42 × 10^8^ (11)	5.77 × 10^8^	2.93 × 10^8^ (21)	1.26 × 10^9^ (51)	NS	7.99 × 10^8^ (51)	4.74 × 10^8^ (59)	2.93 × 10^8^ (45)	4.81 × 10^7^ (39)	7.05 × 10^7^ (56)
M17_iBF	3.29 × 10^6^ (5)	5.50 × 10^6^ (12)	1.52 × 10^7^ (7)	8.62 × 10^6^ (16)	2.37 × 10^9^	9.25 × 10^8^ (32)	6.17 × 10^9^ (33)	NS	4.48 × 10^9^ (39)	3.45 × 10^9^ (57)	2.57 × 10^9^ (49)	6.43 × 10^8^ (41)	1.05 × 10^9^ (51)
M18_iBF	2.54 × 10^8^ (10)	2.06 × 10^8^ (7)	2.02 × 10^8^ (6)	1.20 × 10^8^ (13)	3.52 × 10^7^	1.85 × 10^6^ (33)	9.57 × 10^7^ (49)	NS	4.12 × 10^7^ (42)	2.32 × 10^7^ (33)	1.63 × 10^7^ (48)	4.53 × 10^6^ (42)	5.72 × 10^6^ (49)
M19_iBF	1.82 × 10^6^ (9)	1.95 × 10^6^ (8)	1.96 × 10^6^ (5)	1.13 × 10^6^ (11)	2.70 × 10^7^	4.08 × 10^7^ (19)	6.93 × 10^7^ (50)	NS	4.34 × 10^7^ (43)	2.54 × 10^7^ (39)	1.51 × 10^7^ (32)	2.52 × 10^6^ (37)	5.44 × 10^6^ (48)
M20_iBF	1.40 × 10^8^ (7)	1.04 × 10^8^ (10)	9.07 × 10^7^ (7)	4.57 × 10^7^ (12)	2.21 × 10^7^	5.10 × 10^7^ (21)	6.80 × 10^7^ (51)	NS	5.84 × 10^6^ (55)	4.54 × 10^6^ (37)	2.21 × 10^6^ (18)	1.10 × 10^6^ (37)	8.85 × 105 (51)
M21_iBF	9.47 × 10^6^ (17)	9.19 × 10^6^ (21)	6.68 × 10^6^ (10)	2.24 × 10^6^ (11)	1.32 × 10^6^	8.40 × 10^6^ (22)	9.30 × 10^6^ (63)	NS	5.15 × 10^6^ (37)	3.04 × 10^6^ (35)	3.56 × 10^6^ (22)	2.05 × 10^6^ (58)	5.05 × 10^6^ (58)
P_iBF	1.69 × 10^9^ (11)	8.78 × 10^8^ (18)	5.31 × 10^8^ (8)	2.39 × 10^8^ 13)	2.28 × 10^9^	3.82 × 10^8^ (29)	5.82 × 10^9^ (41)	NS	1.05 × 10^9^ (39)	1.23 × 10^9^ (38)	7.56 × 108 (32)	8.86 × 10^7^ (56)	2.33 × 10^7^ (38)
M22_iBF	NF	NF	NF	NF	2.44 × 10^7^	2.92 × 10^7^ (31)	5.78 × 10^7^ (42)	NS	1.98 × 10^7^ (41)	1.09 × 10^7^ (48)	8.65 × 10^6^ (61)	6.08 × 10^5^ (32)	1.75 × 10^6^ (42)

NS no sample available, NF not found.

## Data Availability

The data presented in this study are available in Supplementary Material.

## Supplementary Materials

The following are available online at https://www.mdpi.com/2218-1989/11/2/97/s1, Figure S1: extracted ion currents of the putatively identified metabolites for 4-fluoro-furanylfentanyl, Figure S2: extracted ion currents of the putatively identified metabolites for isobutyrylfentanyl, Figure S3: MS/MS fragmentation spectra of 4-fluoro-furanylfentanyl metabolites and postulated fragmentation pattern, Figure S4: MS/MS fragmentation spectra of isobutyrylfentanyl metabolites and postulated fragmentation pattern.
