# Supplementary figures and images for: Untargeted Metabolic Profiling of 4-Fluoro-Furanylfentanyl and Isobutyrylfentanyl in Mouse Hepatocytes and Urine by Means of LC-HRMS
Source: Metabolites. 2021 Feb 10;11(2):97. doi: 10.3390/metabo11020097 (PMC7916627; doi:10.3390/metabo11020097)

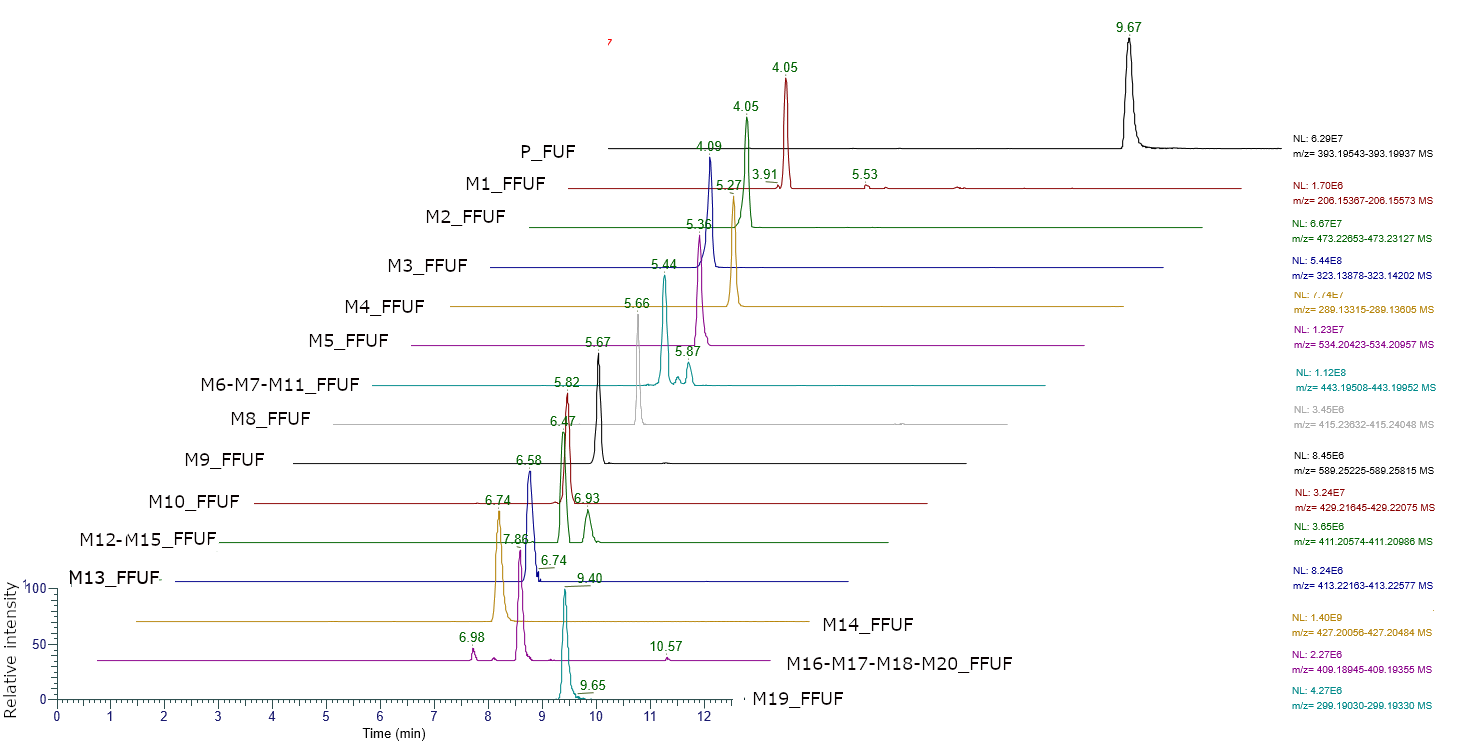

Supplement: Supplementary file 1 [file metabolites-11-00097-s001.zip › Figure S1.png]

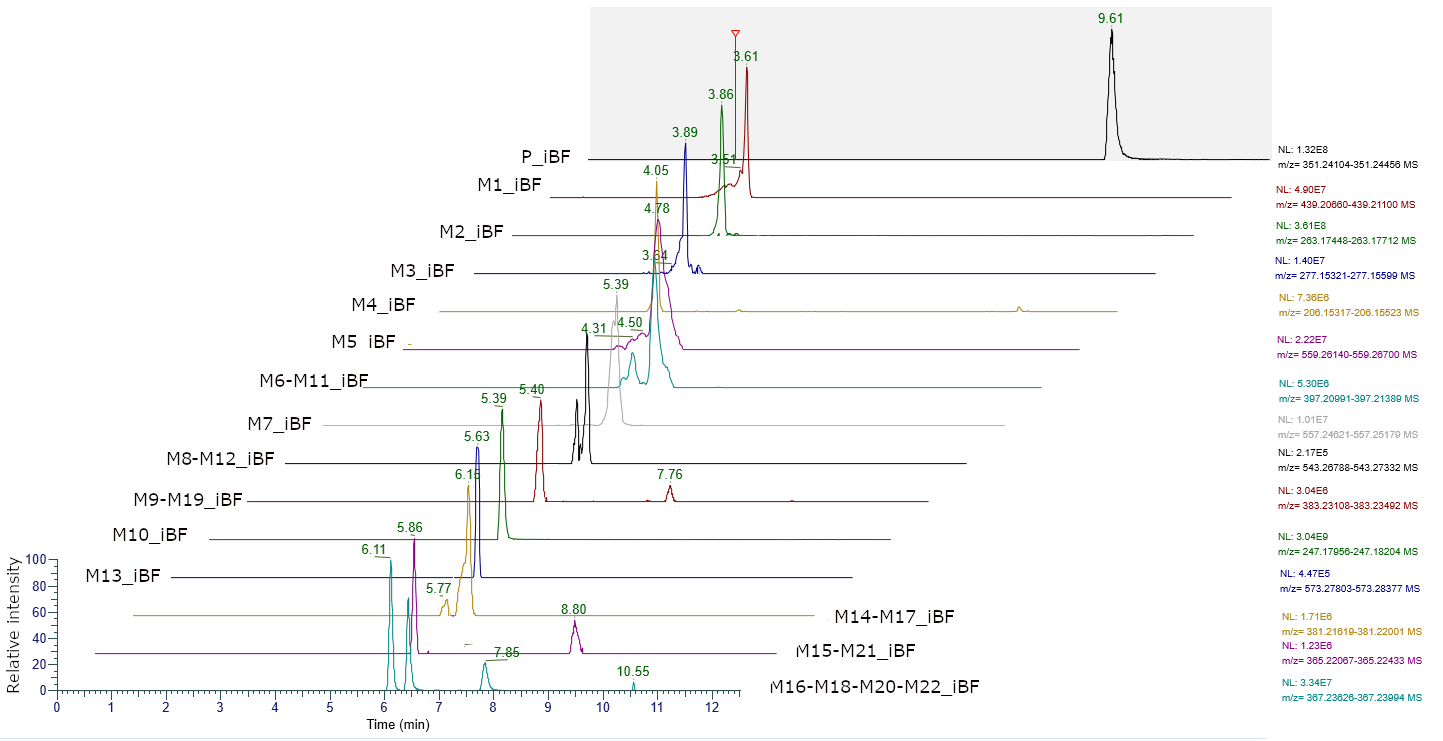

Supplement: Supplementary file 1 [file metabolites-11-00097-s001.zip › Figure S2.png]

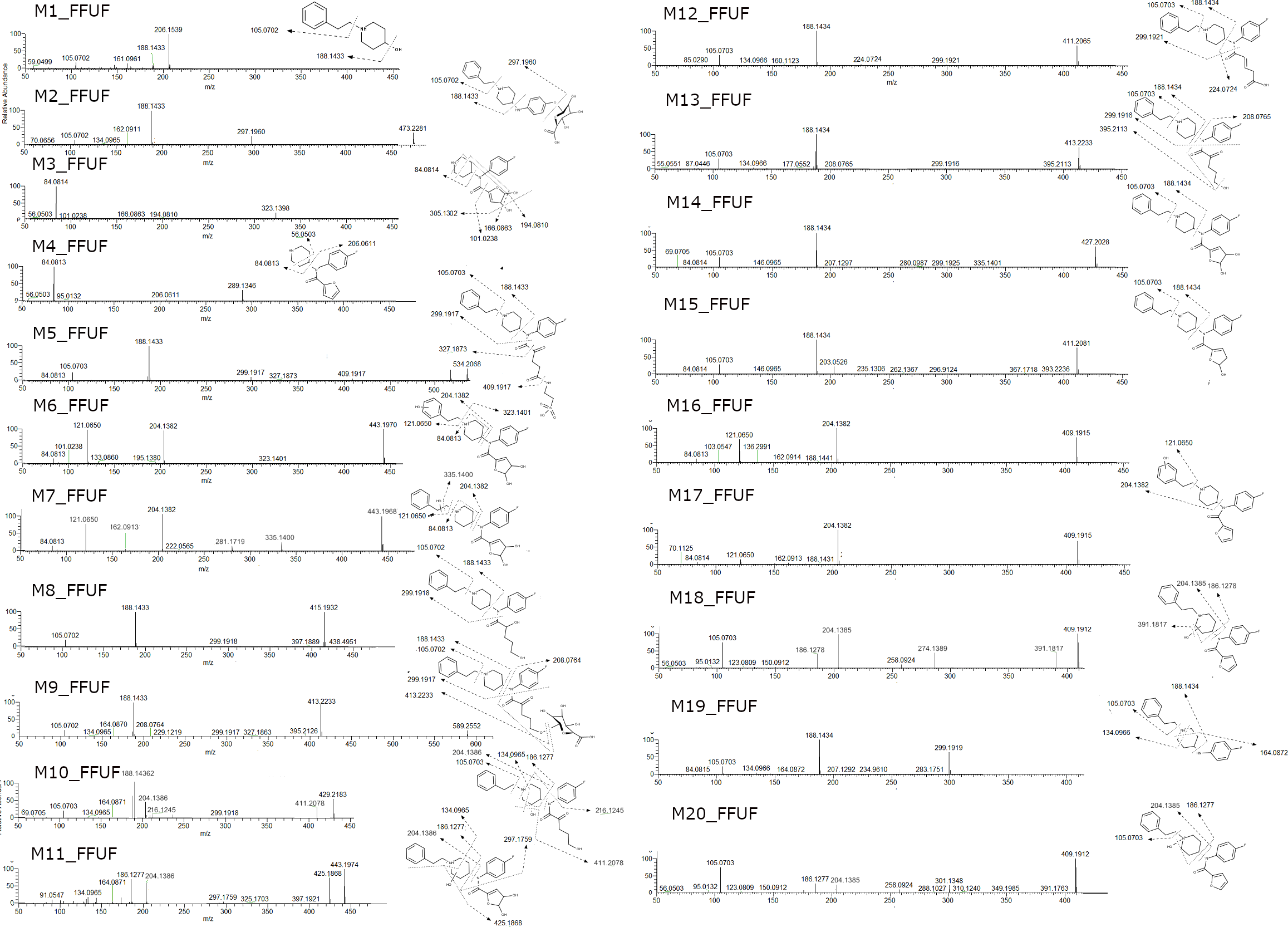

Supplement: Supplementary file 1 [file metabolites-11-00097-s001.zip › Figure S3.png]

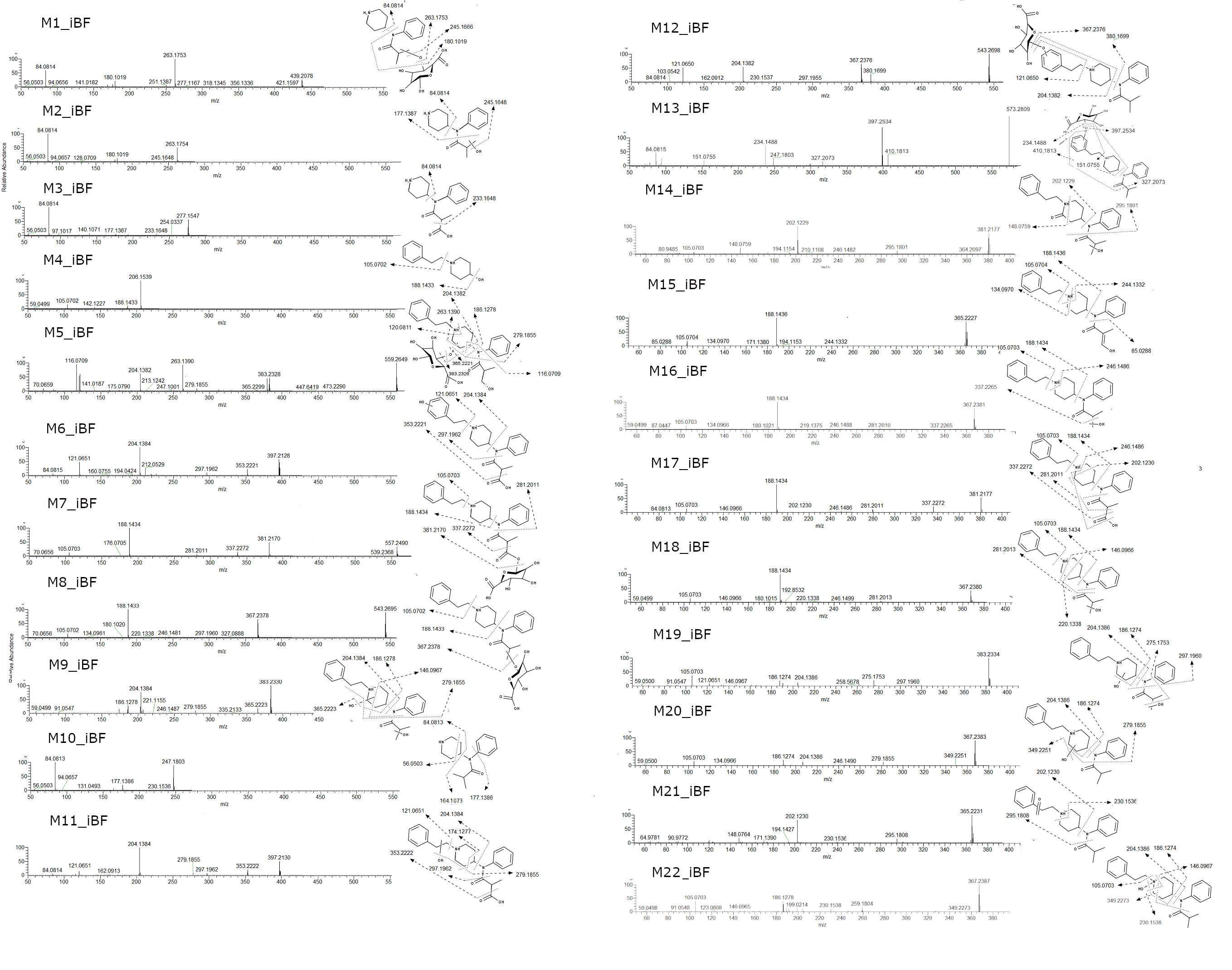

Supplement: Supplementary file 1 [file metabolites-11-00097-s001.zip › Figure S4.png]
